# Meaningful tourism experiences and the cultivation of wellbeing effects: transformative practice of posttraumatic travel

**DOI:** 10.3389/fpsyg.2026.1714606

**Published:** 2026-01-30

**Authors:** Lijun Liu, Pian Pu

**Affiliations:** School of Tourism and Culture Industry, Sichuan Tourism University, Chengdu, China

**Keywords:** meaningful tourism experiences, positive personality traits, tourist transformation, transformative tourism, trauma, wellbeing

## Abstract

**Introduction:**

Travel following major traumatic events can serve as a catalyst for meaning reconstruction and enhanced wellbeing; however, empirical evidence supporting this relationship remains limited. This study examines how reflective and meaningful tourism experiences foster gratitude and multidimensional wellbeing in the post-pandemic context.

**Methods:**

A sequential mixed-methods design was employed. Study 1 involved semi-structured interviews with 20 tourists to examine the psychological impacts of prolonged pandemic-related trauma and to identify meaningful tourism experiences. Data were analyzed using reflexive thematic analysis. Building on these qualitative insights, Study 2 developed and validated a scale measuring meaningful tourism experiences, and subsequently administered a survey to 714 respondents. Partial least squares structural equation modeling (PLS-SEM) with bootstrapping was used to test the relationships among meaningful tourism experiences, gratitude, tourism wellbeing, and overall wellbeing.

**Results:**

Study 1 identified seven themes associated with pandemic-related trauma and tourism experiences. Among them, a core theme of meaningful tourism experiences comprised five dimensions: pleasure, freedom, personal growth, creating memories, and examining life. Study 2 confirmed this five-dimensional structure and demonstrated that such experiences significantly enhanced both eudaimonic and hedonic tourism wellbeing, as well as state gratitude. Eudaimonic tourism wellbeing and state gratitude mediated the effects on both subjective and psychological wellbeing, whereas hedonic tourism wellbeing mediated only subjective wellbeing. Trait gratitude also positively predicted wellbeing outcomes.

**Discussion:**

This study advances the understanding of tourism as a pathway to enhancing wellbeing after collective trauma and offers both theoretical and practical implications for designing transformative experiences.

## 1 Introduction

Although they may leave indelible scars, traumatic events or challenging life circumstances can lead to positive personal growth or transformation (Janoff-Bulman, 1992; Tedeschi et al., 2018). As a profound collective trauma event, the COVID-19 pandemic significantly challenged individuals' core assumptions, threatened their understanding of the meaning of life, and undermined their wellbeing (Bhalla et al., 2021; Cheng and Liu, 2022). Positive psychology suggests that trauma provides opportunities for reflection, deepens an individual's understanding of the fragility of life, and stimulates positive personality traits toward posttraumatic growth and flourishing (Wong, 2020; Zhang et al., 2024). In this process, individual reflection, pursuit, and reestablishment of meaning in life are crucial for coping with trauma and achieving transformation (Frankl, 1992; Liu et al., 2023; Wong et al., 2023). However, our current understanding of the impact of the COVID-19 trauma on people's conception of meaning in life and how to move toward transformation in the wake of such trauma remains insufficient.

Some scholars posit that tourism can assist individuals who have experienced trauma in their search for new meaning in life, helping them rebuild their worldview and facilitating positive change and development (Sengupta, 2022; Wong et al., 2023). Within this context, meaningful tourism experiences refer to travel experiences that encourage reflection on life purpose and foster a sense of personal significance and growth (Câmara et al., 2023; Chirakranont and Sakdiyakorn, 2022). Specifically, meaningful tourism experiences are effective for posttraumatic growth, allowing tourists to alleviate existential crises, reduce psychological distress, and achieve healing and growth (Miao et al., 2021). In addition to satisfying tourists' quest for meaning in life following loss and adversity, meaningful tourism experiences can provide the conditions needed for cultivating a better understanding of life, engaging in self-discovery, and achieving transformation (Chirakranont and Sakdiyakorn, 2022; Sheldon, 2020). This process aligns with the concept of tourist transformation, defined in the literature as positive changes in attitudes, values, and life orientations that result from sustained engagement with meaningful tourism (Kirillova and Lehto, 2015; Pung et al., 2020). Such transformation is regarded as an ongoing process rather than an endpoint (Zhuo et al., 2024). However, current research has paid insufficient attention to meaningful tourism experiences with transformative potential following trauma, and there is a lack of consensus regarding the elements involved in awakening meaning in life during tourism experiences. Therefore, a qualitative investigation is needed to uncover how meaningful tourism experiences emerge following trauma and to delineate their constituent dimensions, thereby advancing the theoretical understanding of tourist transformation.

The trauma caused by the pandemic has sparked individuals' desire for transformation through meaningful tourism while elevating their awareness of wellbeing (Huang et al., 2023). Wellbeing is considered the ideal state of human existence and the cornerstone of a good life (Kay Smith and Diekmann, 2017). Previous studies have shown that meaning in life is closely related to wellbeing (King and Hicks, 2021). The benefits of deriving meaning in life through tourism or leisure activities include healing from trauma and enhancing wellbeing (Iwasaki, 2008; Jain et al., 2023). However, despite the rapidly growing interest in wellbeing among tourism researchers, there is a lack of empirical research on the impact of posttraumatic meaningful tourism experiences on wellbeing. Moreover, according to top-down theories, wellbeing arises from the internal traits that enable individuals to interpret their experiences in a positive way (Diener and Ryan, 2009). Notably, struggling with challenging traumatic events may motivate people to acquire positive personality traits (Cheng and Liu, 2022; Liu et al., 2023). Accordingly, a quantitative approach is essential to empirically examine the combined effects of meaningful tourism experiences and positive personality traits on wellbeing.

Building on these considerations, this study adopts a sequential mixed-methods design to achieve three key objectives, integrating qualitative interview data in the exploratory phase and quantitative survey data in the subsequent phase. First, this study conducts a thematic analysis of interview texts to explore tourist trauma and transformation during the COVID-19 pandemic from a positive psychology perspective. Second, this study employs a mixed-methods approach to identify and validate the dimensions of meaningful tourism experiences with transformative potential. Finally, based on the qualitative research findings and literature review, this study constructs a theoretical framework to examine the effects and mechanisms through which meaningful tourism experiences and positive personality traits can influence wellbeing in the wake of the pandemic.

## 2 Literature review

### 2.1 Traumatic events and transformation

A traumatic event can be defined as an extraordinary and existentially threatening event experienced by an individual that poses a threat to their self-protection and existence (Tedeschi et al., 2018). As urgent experiences that trigger an individual's awareness of “nonbeing,” traumatic events can prompt self-reflection and meaning-making, making them catalysts for personal growth and transformation (Cheng and Liu, 2022; Yalom, 1980). Previous studies have provided evidence of transformation in individuals' cognitive, emotional, and behavioral domains (Liu et al., 2023; Tedeschi et al., 2018). Significantly, moving from adversity to transformation requires people to make adjustments to significant aspects of their lives and reprogram their existence by building new structures of meaning (Janoff-Bulman, 1992). In this respect, seeking and cultivating meaning is a crucial way of coping with trauma and constructively confronting existential anxiety, which ultimately leads to change and flourishing (Frankl, 1992; Janoff-Bulman, 1992; Steger et al., 2011).

Experiences with transformational qualities are not only found in unanticipated liminal experiences and traumas (Liu et al., 2023) but may also occur in diverse service contexts (Neuhofer, 2024). Certainly, the idea that tourism has transformative potential has been widely discussed by researchers (Kirillova et al., 2017a; Sheldon, 2020). Transformative tourism experiences are defined as experiences that can deeply impact tourists, guiding them to reflect upon and increase their awareness of their own existence, ultimately leading to positive changes in their personal attitudes, values, and behaviors (Pung et al., 2020). Tourism scholars have conducted research on the theoretical frameworks of transformative experiences, as well as their triggers, influencing factors, processes, mechanisms, and outcomes (Milazzo and Soulard, 2024), exploring various types of tourism that may trigger transformation and growth.

Of course, while travel can be transformative, not every experience prompts positive change for tourists (Brown, 2013). Highly meaningful tourism experiences and the availability of threshold tourism spaces for developing self-insight are considered important for catalyzing transformation (Kirillova and Lehto, 2015; Neuhofer, 2024). Scholars further emphasize that tourist transformation is a continuous process (Zhuo et al., 2024). Significantly, tourism experiences that enhance life's significance are effective means of realizing the goal of tourism to have a profound impact on tourists' change in the long term (Chirakranont and Sakdiyakorn, 2022; Kirillova et al., 2017a).

Such insights notwithstanding, relatively few studies link traveler trauma to transformation, with those that do often focusing on two main aspects. First, some researchers have focused on travel as a means of posttraumatic healing and an effective motivator for travelers to engage in self-reflection, growth, and transformation (Bhalla et al., 2021; Sengupta, 2022). In this respect, scholars have captured the positive effects of traveling after the COVID-19 pandemic in inspiring travelers to emerge from darkness and hardship to gain spiritual strength and transformation (Buckley and Westaway, 2020). Second, others have examined how encountering trauma or trauma-like adversities—such as challenges, culture shock, and disorienting dilemmas during travel can result in transformation or posttraumatic growth (Kirillova et al., 2017b; Liu et al., 2023). Accordingly, this study argues that the COVID-19 pandemic, as a collective traumatic experience, provides a critical context for advancing the understanding of travelers' trauma and their potential for transformation.

### 2.2 Meaningful tourism experiences

Tourism has great potential as an experience that enriches the meaning of human life (Câmara et al., 2023; Wattanacharoensil et al., 2024). Meaning in life refers to the extent to which an individual's life is meaningful, guided and motivated by valuable goals, and significant to the world (George and Park, 2016). Comprehension, purpose, and existential importance have been identified as three important dimensions that make up meaning in life (Martela and Steger, 2016). Numerous studies have shown that meaning in life is strongly associated with a range of positive and healthy indicators, such as trauma coping, happiness, wellbeing, and life satisfaction, while a lack of meaning is linked to negative psychological conditions like boredom, anxiety, and even existential emptiness (King and Hicks, 2021; Steger et al., 2011).

Câmara et al. (2023, p. 18) characterized meaningful tourism experiences as holistic and metaconceptual, positing that such experiences entail a multifaceted process involving personal development, emotional growth, increased wellbeing, behavioral development, and relationship building. Drawing on the theory of meaning in life, Jain et al. (2023) defined the consumption of specific categories, such as tourism or processes, as meaning-oriented consumption. They argued that meaningful consumption creates meaning by providing consumers with a sense of understanding, purpose, and significance, which have emotional, health, behavioral, and attitudinal impacts (Jain et al., 2023). Based on the above-discussed definitions, this study defines meaningful tourism experiences as experiences that offer tourists a sense of meaning in life—that is, experiences that inspire tourists to understand and appreciate the meaning of life and to cultivate their awareness of the purpose, mission, and significance of their own lives. Over time, meaningful tourism experiences can promote the development and transformation of tourists (Câmara et al., 2023).

In the field of tourism, only a handful of studies have focused directly on meaningful tourism experiences, the majority doing so from the perspective of how destinations drive and provide meaningful experiences (Chirakranont and Sakdiyakorn, 2022). For instance, Wattanacharoensil et al. (2024) identified destinations that promote the meaningful experiences of key stakeholders and introduced six essential dimensions of a meaningful destination. Some scholars have focused on the meaning of life in tourism (Bosangit et al., 2015), while others have considered it as a precursor variable for explaining tourists' intentions and behaviors (Wong et al., 2023). Consequently, although the value of meaningful experiences is widely recognized, the specific components of such experiences and their functioning within posttraumatic contexts remain insufficiently explored.

### 2.3 Wellbeing

Drawing on eudemonic and hedonic philosophy, psychologists generally classify wellbeing into eudaimonic wellbeing and hedonic wellbeing (Ryan and Deci, 2001). Eudaimonic wellbeing emphasizes self-actualization and meaning, reflecting the degree to which a person fully unleashes their potential (Huta and Waterman, 2014). Among the various theories supporting eudaimonic wellbeing, Ryff's (1989) six-dimensional model of psychological wellbeing is among the most widely applied. Meanwhile, hedonic wellbeing focuses on the experience of pleasure and regards wellbeing as the maximization of pleasure and avoidance of pain (Diener et al., 2003). In this regard, subjective wellbeing, which reflects a person's overall assessment of all aspects of their life, typifies hedonic wellbeing (Diner, 1984). To elucidate the nature of wellbeing, a growing number of scholars have advanced the need for equal consideration of both subjective and psychological wellbeing (Ryan and Deci, 2001).

Wellbeing can be measured at either the trait or state level (Huta and Waterman, 2014). At the trait level, wellbeing typically refers to the individual's relatively stable, typical, or average degree of hedonic and eudaimonic wellbeing. At the state level, wellbeing reflects the degree of happiness experienced by an individual at a specific point in time, over a particular period, or during a specific activity (Huta and Waterman, 2014; Peng et al., 2023). For example, Ryff (1989) Psychological Wellbeing Scale and Diener et al.'s (1985) Life Satisfaction Scale typically assess an individual's overall wellbeing at the trait level. Meanwhile, Waterman's (1993) Personally Expressive Activities Questionnaire (PEAQ) can be used to measure wellbeing at the state level when individuals engage in self-defining activities.

There are two opposing theoretical perspectives on wellbeing: the top–down theory and the bottom–up spillover theory. The former posits that wellbeing is not derived from objective circumstances but from intrinsic traits that facilitate an individual's positive interpretation of their experiences. In contrast, the latter emphasizes that a happy life is the accumulation of pleasurable moments, and overall wellbeing comes from the sum of happiness experienced across various important life domains (Diener and Ryan, 2009).

Relevant tourism research has primarily focused on the impact of tourism activities on wellbeing (Su et al., 2020), notations and dimensions of tourist wellbeing (Filep et al., 2022), and the factors influencing wellbeing as well as outcomes and benefits thereof (Vada et al., 2020). In addition, scholars have tended to prefer flow, self-determination, and bottom-up spillover theories (Sirgy, 2019). However, the wellbeing that arises from specific tourism situations has frequently been overlooked. Indeed, scholars have primarily focused on wellbeing at the trait level as an outcome variable for the tourism experience, with fewer studies examining wellbeing at the state level (Lee and Jeong, 2021). This oversight needs to be addressed, particularly insofar as it may hinder our understanding of wellbeing. To bridge these theoretical and empirical gaps, this study proposes and examines an integrative model in which posttraumatic meaningful tourism experiences (a bottom-up factor) and trait gratitude (a top-down factor) jointly shape both state and trait wellbeing.

Accordingly, this study employed subjective wellbeing and psychological wellbeing to measure overall wellbeing at the trait level, while evaluating wellbeing at the state level through tourism wellbeing. The latter can be defined as the happiness experienced by tourists during their participation in tourism activities and comprises both eudaimonic and hedonic tourism wellbeing (Su et al., 2021). Eudaimonic tourism wellbeing refers to the happiness with which people fully engage in travel activities, recognizing the opportunity to express their authentic selves and achieve their full potential (Lee and Jeong, 2021). Hedonic tourism wellbeing describes the joy and pleasure experienced by tourists whose needs are satisfied during tourism activities (Park and Ahn, 2022; Waterman, 1993).

## 3 Methodology

**Figure 1:**
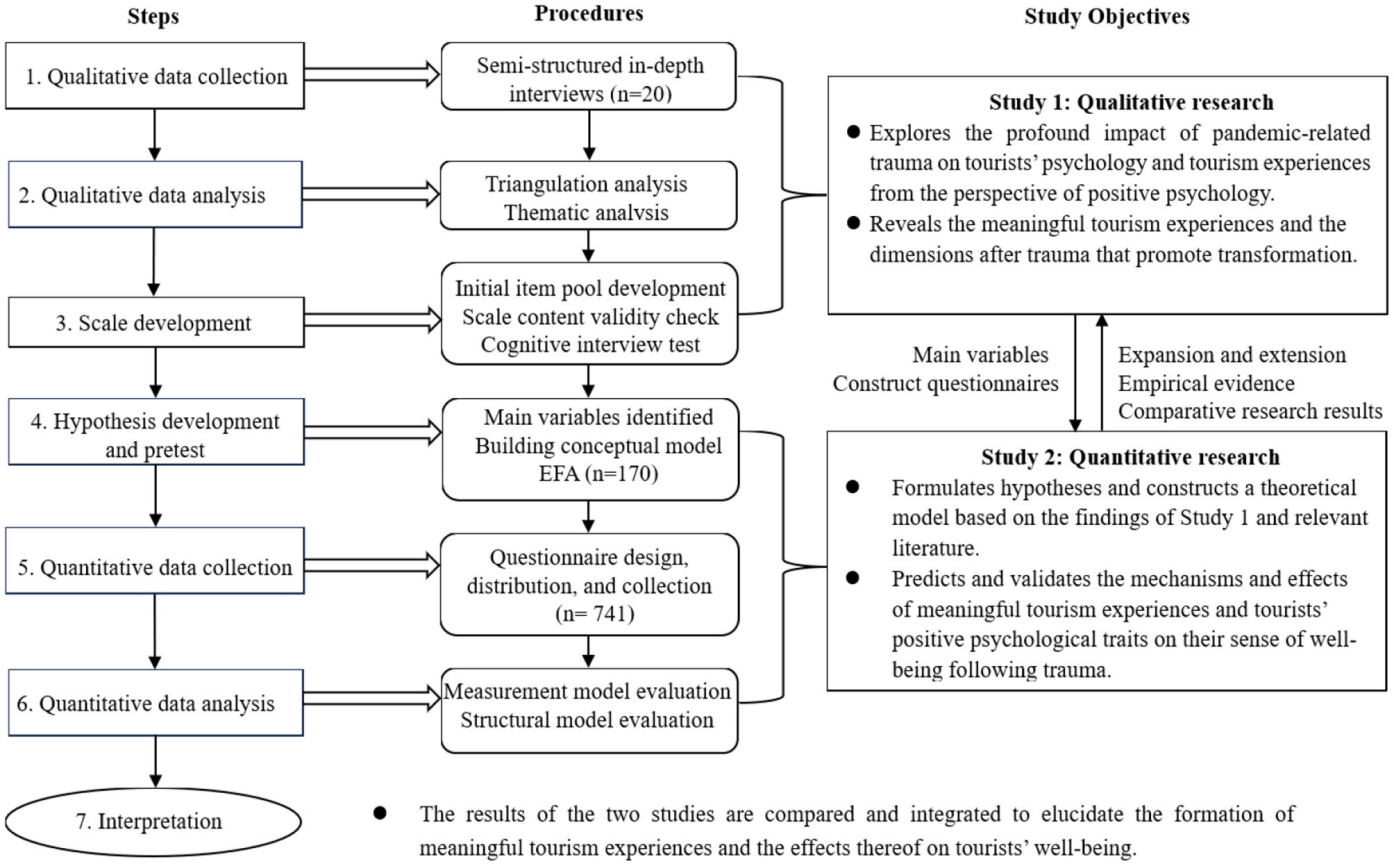
Overview of the research process.

This study utilized an ordered mixed-methods design that combines qualitative and quantitative approaches (Figure 1). First, adopting a positive psychology perspective, Study 1 explored the profound impact of the three-year pandemic trauma on tourists' psychology and tourism experiences. In doing so, it identified meaningful tourism experiences and the dimensions that promote transformation after trauma. Second, drawing on the literature, findings of Study 1, and questionnaire data, Study 2 constructed a theoretical model to predict and validate the mechanisms and effects of posttraumatic meaningful tourism experiences and positive psychological traits on wellbeing. More specifically, Study 1 employed semi-structured interviews with 20 purposively selected participants, whereas Study 2 conducted a questionnaire survey using both online and field data collection methods, which yielded 714 valid responses. The theoretical model in Study 2 examines how meaningful tourism experiences and trait gratitude affect wellbeing, with state gratitude and tourism wellbeing acting as mediating variables. As such, the qualitative study served as the foundation for the subsequent quantitative study, helping to identify variables and construct the questionnaire, with the second study constituting an expansion and extension of Study 1 (Creswell and Clark, 2018). Comparison and integration of the results of both sub-studies elicited valuable insights into posttraumatic meaningful tourism experiences and wellbeing effects.

## 4 Study 1: qualitative research

### 4.1 Materials and methodology

On January 8, 2023, China officially lifted the Category A infectious disease prevention and control measures for COVID-19, allowing travelers' daily lives and movements to proceed freely. This marked a turning point after nearly three years of strict pandemic control in China, which included nationwide nucleic acid testing, travel quarantines, and other stringent measures. Therefore, this study collected qualitative data from January to March 2023 using purposive sampling.

Study 1 adopted a hermeneutic paradigm and used semi-structured interviews as its primary method of data collection and analysis. The semi-structured interviews adhered to an outline. The initial outline was drafted based on a review of the literature, revised and adjusted based on interviews with two participants, and subsequently finalized based on the feedback of over 20 master's and doctoral students in the field of tourism. Interviews comprised four components. First, the discussion focused on the impact of the pandemic on tourists' work, lifestyle, and travel as well as the transformations it caused. The second part focused on tourism choices and experiences after the end of the pandemic. The third component concerned tourists' interpretations of meaningful tourism experiences that contributed to their transformation. The final section collected the interviewees' basic information. Details of the outline are provided in Appendix A.

To obtain a more in-depth and detailed interpretive understanding, participants were selected based on the following criteria: (a) participant was 18 years of age or older, (b) had a travel frequency of two or more trips per year before the pandemic, and (c) had engaged in more than two days of overnight tourism in 2023. Ultimately, semi-structured interviews were conducted with 20 tourists, T01–T20. Of the total participants, 12 were interviewed face-to-face in Chengdu, China, while the remaining 8 were interviewed online via Tencent Meeting, a popular audio and video conferencing tool in China. Each interview lasted approximately 40–90 minutes. Table 1 presents the interviewees' basic information. All interviews were audio-recorded with participants' consent, transcribed immediately post-interview, and subsequently imported into NVivo 14 for data analysis. Data collection and analysis were conducted simultaneously in Chinese. This study adopted reflective thematic analysis, as this allows the researcher to analyze the text in a highly flexible, creative, and interpretive way based on reflection on the data and issues (Braun and Clarke, 2019). Guided by Braun and Clarke's (2006, 2019) theoretical approach, data analysis involved six stages.

**Table 1:** Demographic profile of interviewees.

**NO**.	**Gender**	**Age**	**Education**	**City**
T01	Male	41	Bachelor's	Chengdu
T02	Male	20	Bachelor's	Urumqi
T03	Female	24	Master's	Chengdu
T04	Female	37	Master's	Dazhou
T05	Female	32	Master's	Chengdu
T06	Female	31	Doctorate	Chongqing
T07	Male	28	Master's	Chengdu
T08	Male	28	Bachelor's	Nanchang
T09	Male	53	Doctorate	Urumqi
T10	Female	60	Bachelor's	Chengdu
T11	Female	18	Bachelor's	Beijing
T12	Female	58	High School	Chengdu
T13	Male	65	High School	Chengdu
T14	Female	32	Master's	Chengdu
T15	Male	33	Bachelor's	Chengdu
T16	Male	36	Master's	Zhejiang
T17	Male	46	Associate's	Hangzhou
T18	Female	45	Bachelor's	Chongqing
T19	Female	21	Bachelor's	Suining
T20	Male	62	Associate's	Chongqing

Systematic strategies to ensure methodological rigor were implemented throughout all phases of the study: design, data collection, and analysis. First, the research design was rigorously developed based on a comprehensive literature review, expert consultations, and a pilot study that incorporated participant feedback. Second, consistent with the methodological stance of reflexive thematic analysis, which emphasizes the interpretive generation of meaning, the sampling process followed the principle of information power (Malterud et al., 2016). Data collection continued until interviews 17 to 20 yielded no new substantive themes and existing themes were merely deepened, suggesting that theoretical sufficiency had been attained (Braun and Clarke, 2021). To further ensure trustworthiness, multiple triangulation strategies were employed: (1) Investigator triangulation: Two researchers independently coded the data, resolving discrepancies through iterative discussions until consensus was reached. (2) Data/context triangulation: Data were collected from both face-to-face and online video interviews to strengthen the contextual robustness of the findings. (3) Independent audit: An external expert, not involved in the initial analysis, systematically reviewed the entire analytic process and coding decisions to ensure objectivity and transparency (Decrop, 1999). Furthermore, all analyses were conducted using NVivo software, and a comprehensive audit trail was maintained that documented the complete decision-making process from raw data to thematic development, thereby ensuring the dependability and confirmability of the research process.

### 4.2 Results

**Figure 2:**
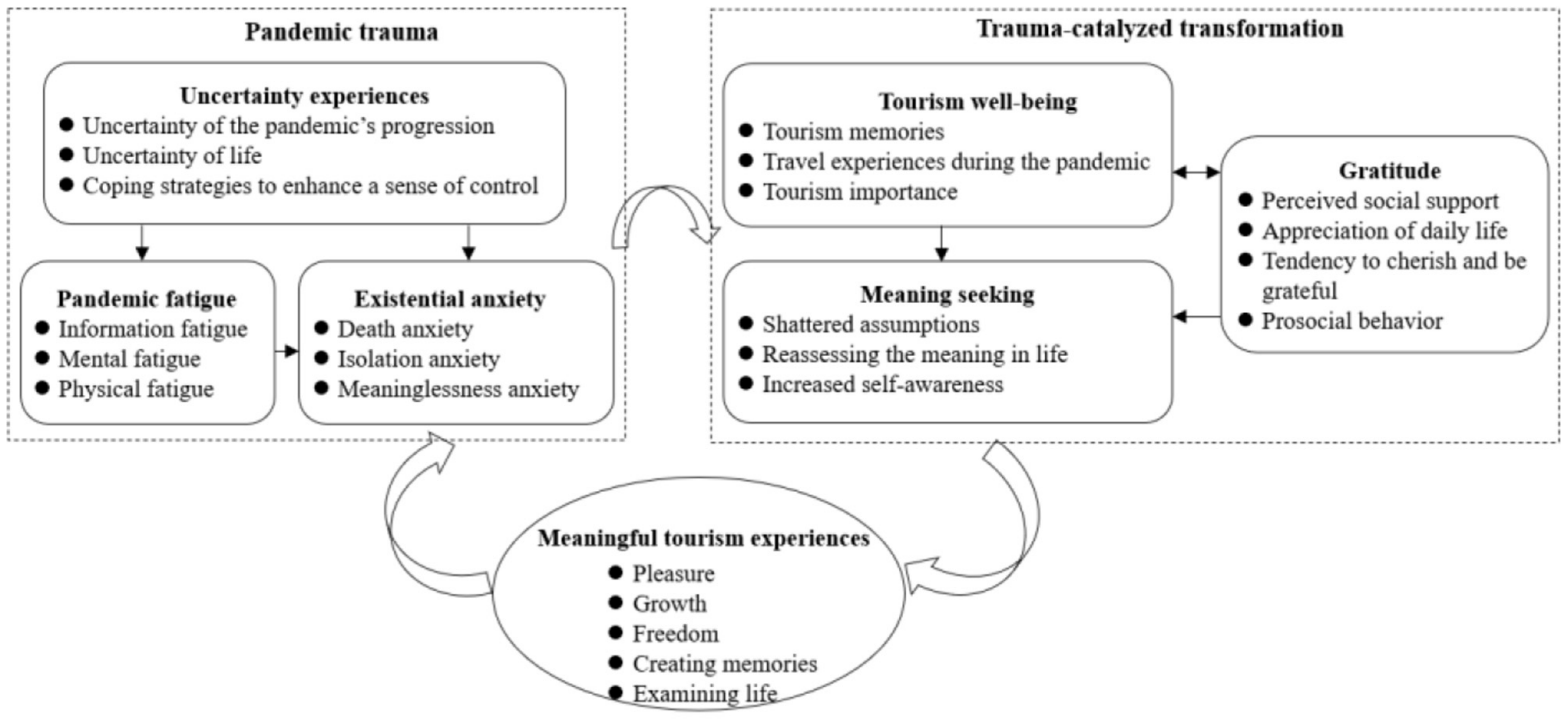
Thematic relationship diagram.

A total of 24 sub-themes and 7 themes were generated through inductive analysis, synthesis, and comparison. The themes comprised uncertainty experience, pandemic fatigue, existential anxiety, meaning-seeking, gratitude, tourism wellbeing, and meaningful tourism experiences. The corresponding sub-themes and connotations are provided in Appendix B. The first three themes reflect the collective trauma experiences of tourists during the COVID-19 pandemic. Specifically, the themes of meaning-seeking, gratitude, and tourism wellbeing address the growth and transformation triggered by trauma. Furthermore, meaningful tourism experiences were found to comprise five dimensions positively associated with meaning in life: pleasure, freedom, growth, creating memories, and examining life. Figure 2 illustrates the relationships between the themes.

### 4.3 Discussion

#### 4.3.1 Pandemic trauma and transformation

The sustained, cumulative pressure of the COVID-19 pandemic made it a widespread collective trauma event. In this respect, the theme of *uncertainty experience* reflected the various uncertainties tourists experienced in terms of potential losses and threats—including physical harm, mental health issues, wasted time, and financial crises—as well as the efforts made to cope with these uncertainties. This pervasive uncertainty fundamentally shattered individuals' assumptive worlds (Janoff-Bulman, 1992), eroding their sense of safety, predictability, and control. Facing this vague and intangible threat, participants reported experiencing *pandemic fatigue* and, to some extent, *existential anxiety*. As articulated by participants, “The most frightening thing is the recurrent nature of the pandemic... never knowing when it will end” (T07), and “Every day felt anxious and irritable... I haven't felt truly relaxed over the past few years” (T20). Pandemic fatigue contributes to the likelihood of tourists feeling emotionally drained and physically exhausted, which may evolve into feelings of hopelessness, loneliness, and anxiety about the future. Significantly, although pandemic fatigue may have gradually faded with the end of the pandemic, the sense of meaninglessness it caused may have had a more profound and lasting effect on tourists. Traumatic experiences during the COVID-19 pandemic—such as the threat of death, constant vigilance, and the loss of daily routines—exposed people to existential dilemmas, leading to existential anxiety related to death, loneliness, and meaninglessness among tourists. This was evidenced by the observation that nearly all participants explicitly confronted the topic of death and engaged in deep reflection on their own existence and the meaning of life.

While pandemic trauma greatly threatened people's conception of meaning in life and undermined their wellbeing, it also provided the conditions necessary to foster individual transformation and growth. First, due to factors such as travel restrictions, participants reported becoming acutely aware of the importance and value of tourism in their lives. Tourism memories, imperfect travel experiences during the pandemic, and their perceptions of the importance of tourism further reinforced their understanding of *tourism wellbeing*. For example, one participant reflected, “I often look back at photos from previous trips, especially missing the days when I could travel freely” (T01). Another noted, “If possible, I try to travel whenever I can, even if just for a day or two, and it makes me feel very happy” (T05). Second, the COVID-19 pandemic prompted tourists to cherish and appreciate what they have and recognize the beauty and preciousness of life, encouraging their sense of *gratitude*. This finding aligns with previous research on posttraumatic growth (Cheng and Liu, 2022; Tedeschi and Calhoun, 2004). Enhanced gratitude can be observed in three levels and two types (state and trait): emotional responses elicited by perceiving support, help, and sacrifices from others (state gratitude); immediate emotional reactions of gratitude prompted by appreciation of aspects of life that are worthy of attention and care (state gratitude); and a broader life orientation trait, reflecting an appreciation and gratitude toward life, the world, and others (trait gratitude).

Finally, the COVID-19 trauma significantly disrupted people's previous assumptions that the future is predictable, controllable, and secure. Reflection and reassessment of the meaning in life, accompanied by increased self-awareness (e.g., self-care, respect for one's inner self, and self-connectedness) can help tourists better understand the essence of life and its meaning. Thus, constructively confronting life's finite and fragile nature allows tourists to begin to re-establish their understanding of life's meaning, significance, and purpose. As one interviewee reflected, “The pandemic has given me much to ponder over these years... the most important thing is to live in the present and make every day count” (T11). Therefore, *meaning-seeking* and *tourism wellbeing* can form a foundation for tourism activities to enrich tourists' meaning in life following trauma.

#### 4.3.2 Dimensions of meaningful tourism experiences

Thematic analysis revealed that meaningful tourism experiences comprise five dimensions. First, the *pleasure dimension* reflects the positive emotions that tourists experience during their travel, including happiness, joy, relaxation, and enjoyment, and is considered a core aspect of meaning in life. As noted by King and Hicks (2021), individuals are more likely to derive meaning from experiences that elicit positive emotions. Consistent with this perspective, many participants emphasized that “the most important function of travel is to make oneself happy” and that “happiness itself is meaningful.” Second, influenced by the limitations and restrictions imposed by the COVID-19 pandemic, the *freedom dimension* refers to tourists' desire for a life and travel experiences free from constraints. This desire is rooted in the fundamental human need for autonomy, the fulfillment of which is essential for optimal functioning and wellbeing (Ryan and Deci, 2001). Accordingly, post-crisis travel functioned as a powerful compensatory mechanism that restored tourists' sense of agency and control, enabling them to break free from the constraints of everyday life and regain a sense of meaning.

Third, the *growth dimension* reveals that the tourism experience provides an important environment for acquiring knowledge, improving skills, broadening the mind, and exploring self-potential, thus laying the foundation for self-actualization. The growth tourism experience can prompt travelers to develop new goals and seek and create new meaning. Fourth, the *creating memories dimension* offers a sense of meaning to tourists in two respects: the continuity of time and the maintenance of intimate relationships. Tourism memory can enhance tourists' understanding of time continuity and life coherence related to the past, present, and future. Meanwhile, intimate relationships provide meaning because the existence of close-knit groups transcends the limitations of the physical body, thereby expanding the boundaries of the self and achieving a form of symbolic immortality (King and Hicks, 2021). As one participant expressed, “Nothing lasts forever. Creating beautiful shared memories with the people I love will give me something meaningful to look back on when I grow old” (T12). Finally, the *examining life dimension* reflects tourists' understanding of and reflection on themselves, others, and the nature of life. This dimension is closely related to the “emergency experience” of the COVID-19 pandemic (Liu et al., 2023) and is inseparable from the threshold characteristics of tourism experiences. Reflection and examination in tourism help individuals reassess life's priorities and rebuild a philosophy of life, thus facilitating their identification of the true meaning of life.

### 4.4 Development of a meaningful tourism experience scale

Following the recommendations of Churchill (1979) and DeVellis (2017), Study 1 developed a meaningful tourism experience scale through a five-stage process. In the first stage, a comprehensive literature review was conducted to define a meaningful tourism experience and extract relevant items to construct an item pool. In the second stage, thematic analysis was conducted to define the five dimensions of meaningful tourism experiences. The researchers captured codes in phrases or sentences from the interview transcripts that reflected the interviewees' perspectives and utilized these codes to expand the item pool. In the third stage, several rounds of assessment were conducted to purify the contents of the item pool, resulting in the development of an initial scale containing 23 items. In the fourth stage, a panel of 20 doctoral and master's students specializing in tourism reviewed the content validity of the scale and provided feedback. The panel identified one item (“During this trip, I experienced moments for self-reflection”) as conceptually redundant with others in the examining Life dimension (e.g., “I reflected on what truly matters in life”) and lacking discriminant validity. To improve scale parsimony and clarity, this item was removed by panel consensus, resulting in a refined 22-item version. Finally, the scale was distributed to the eight tourists interviewed in Study 1, and the items were fine-tuned based on comments.

## 5 Study 2: quantitative research

### 5.1 Main variables

Study 2 integrated bottom–up and top–down theories of wellbeing to explore the effects and mechanisms of posttraumatic meaningful tourism experiences (bottom-up influence) and positive personality traits (top-down influence) on wellbeing. According to Study 1, gratitude is a positive personality trait that can be significantly enhanced after experiencing loss and adversity. A review of the literature indicated that gratitude is not only considered one of the most influential traits on subjective wellbeing (Watkins et al., 2003) but also strongly predicts psychological wellbeing (Măirean et al., 2019; Wood et al., 2009). Therefore, this sub-study selected trait gratitude as a key predictor of wellbeing from a top-down perspective.

The bottom-up spillover theory suggests that wellbeing is the sum of many pleasures in life and is influenced by wellbeing in specific life domains or activities (Sirgy, 2019). Therefore, meaningful tourism experiences and wellbeing were selected as antecedent variables that influenced wellbeing from a bottom-up perspective. It is worth noting that state gratitude is not only triggered by tourism experiences (Westoby et al., 2022) but is also positively associated with an individual's overall wellbeing (Emmons and McCullough, 2003). Accordingly, Study 2 primarily focused on the following variables: meaningful tourism experiences, tourism wellbeing (including eudaimonic and hedonic tourism wellbeing), gratitude (trait and state gratitude), and overall wellbeing (subjective and psychological wellbeing).

### 5.2 Hypothesis development

Previous studies suggest that wellbeing generated in specific tourism situations is closely related to the tourism experience (Lee and Jeong, 2021; Peng et al., 2023). Generally, positive tourism experiences increase tourists' wellbeing, whereas disappointing tourism experiences have a negative impact (Kay Smith and Diekmann, 2017). Park and Ahn (2022) found that the experience of pleasure and detachment during tourism activities positively influences hedonic tourism wellbeing, while self-reflection and personal meaning positively affect eudaimonic wellbeing. In Study 2, meaningful tourism experiences included both sensory pleasures arising from emotional and hedonic aspects, as well as deeper spiritual levels of reflection, self-realization, and personal growth. Therefore, this study proposed the following hypotheses:

H1a: Meaningful tourism experiences have a positive effect on eudaimonic tourism wellbeing.

H1b: Meaningful tourism experiences have a positive effect on hedonic tourism wellbeing.

Based on the bottom–up spillover theory, tourism scholars argue that wellbeing during a single trip plays an important role in the accumulation of overall wellbeing (Peng et al., 2023; Sirgy, 2019). Indeed, some scholars stress that, although a single trip experience has a limited direct impact on a tourist's overall wellbeing, it can still positively impact their overall wellbeing by influencing the wellbeing experienced during the tourism activity (Kay Smith and Diekmann, 2017; Park and Ahn, 2022). In other words, meaningful tourism experiences may improve overall wellbeing by impacting tourism wellbeing. Therefore, this study proposed the following hypotheses:

H2a: Eudaimonic tourism wellbeing mediates the effect of meaningful tourism experience on subjective wellbeing.

H2b: Eudaimonic tourism wellbeing mediates the effect of meaningful tourism experience on psychological wellbeing.

H3a: Hedonic tourism wellbeing mediates the effect of meaningful tourism experience on subjective wellbeing.

H3b: Hedonic tourism wellbeing mediates the effect of meaningful tourism experience on psychological wellbeing.

According to scholars in the field of consumption, consumption can trigger customer gratitude, especially experiential consumption (Walker et al., 2016). Elicited by experiences and stemming from a sense of aimless appreciation, this is a form of gratitude in the broad sense (Adler and Fagley, 2005). In the context of tourism and hospitality, scholars have proposed that positive encounters between tourists and residents (Tu and Ma, 2021), contact and connections between tourists (Filep et al., 2017), and the quality of the experience can influence tourists' state gratitude (Kim and Lee, 2013). Research suggests that both tourism (Westoby et al., 2022), and outdoor recreation (Grimwood et al., 2023) can contribute to the development of gratitude practices that can elevate people's gratitude in the post-pandemic period. Therefore, this study proposed the following hypothesis:

H4: Meaningful tourism experience has a positive effect on state gratitude.

Based on the top–down theory, gratitude is considered an effective way to enhance wellbeing. For example, Watkins et al. (2003) identified trait gratitude as an important emotional trait influencing subjective wellbeing. Adler and Fagley (2005) found that, even after controlling for the effects of traits like optimism, trait gratitude remained significantly associated with life satisfaction and positive affect. Research has also confirmed the positive effects of gratitude on psychological wellbeing (Măirean et al., 2019; Wood et al., 2009). Accordingly, this study proposed the following hypotheses.

H5a: Tourists' trait gratitude has a positive effect on subjective wellbeing.

H5b: Tourists' trait gratitude has a positive effect on psychological wellbeing.

As a positive affective trait, trait gratitude can motivate individuals to cope with problems from a more positive perspective and have greater appreciation for what they have, thereby experiencing more gratitude (Emmons and McCullough, 2003). In other words, people with higher trait gratitude tend to experience more intense state gratitude when they receive benefits (e.g., favor, help, joyful experiences), encounter a higher frequency of gratitude experiences, and feel grateful for a wider range of things. Similarly, Wood et al. (2008) suggested that trait gratitude is a predictor of state gratitude. Therefore, this study proposed the following hypothesis:

H6: Tourists' trait gratitude has a positive effect on state gratitude.

Finally, individuals in a state of gratitude are more satisfied with their overall lives, have closer connections with others, and possess higher levels of wellbeing (Emmons and McCullough, 2003). Amid the uncertainty in the post-pandemic era, some scholars have argued that travel experiences can be used to stimulate feelings of gratitude and thus enhance travelers' wellbeing (Grimwood et al., 2023; Westoby et al., 2022). In other words, meaningful tourism experiences can indirectly affect wellbeing by influencing the state gratitude. Accordingly, this study proposed the following hypotheses:

H7a: State gratitude mediates the effect of meaningful tourism experience on subjective wellbeing.

H7b: State gratitude mediates the effect of meaningful tourism experience on psychological wellbeing.

**Figure 3:**
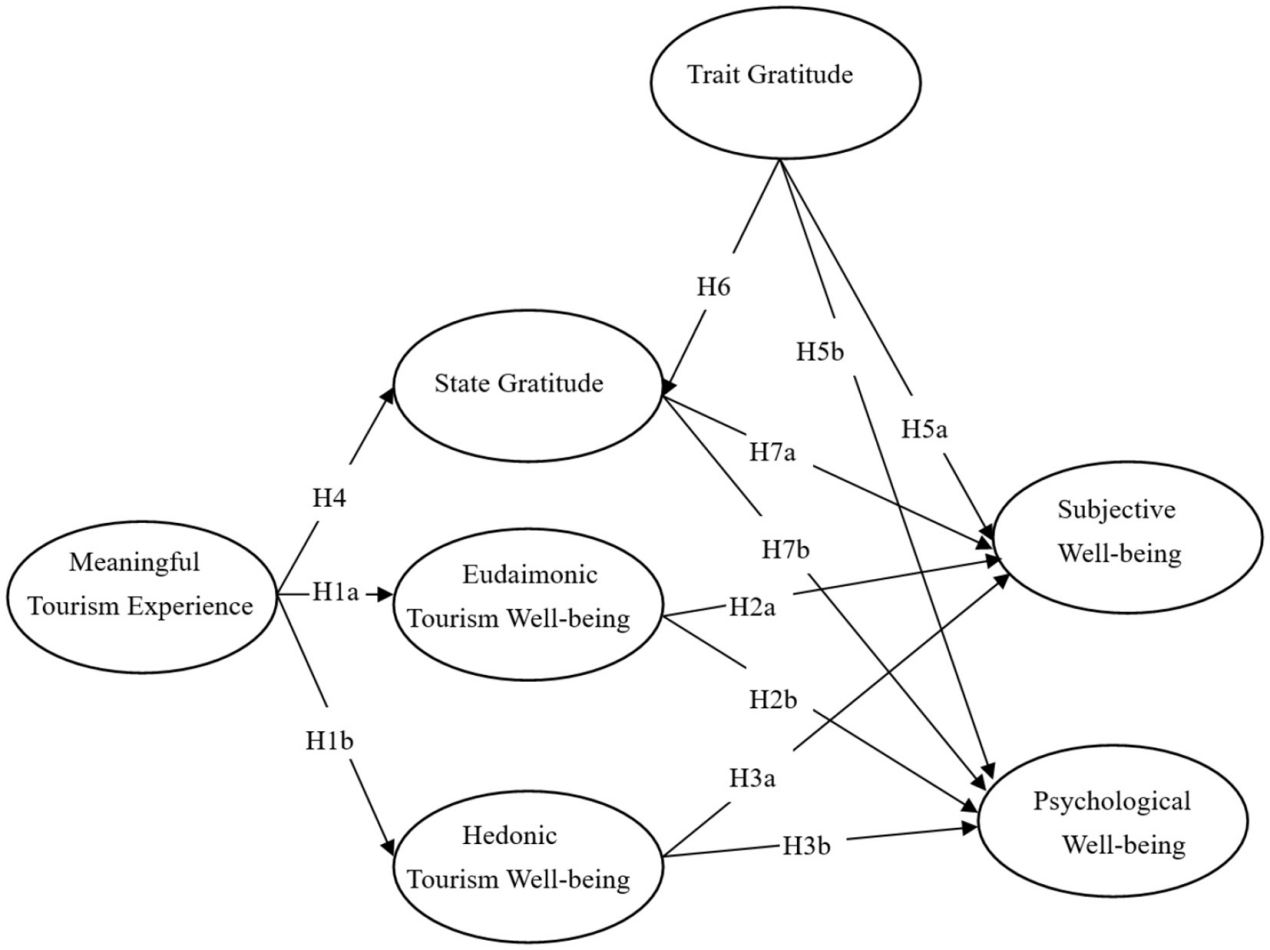
Research framework.

Figure 3 presents Study 2′s research hypotheses and theoretical model.

### 5.3 Data and methodology

#### 5.3.1 Questionnaire design

Apart from the meaningful tourism experiences variable, the items for the other variables examined in this study were based on well-established scales and presented in a Likert 7-point scale format. Study 2 used the 22 items developed in Study 1 to assess meaningful tourism experiences. Tourism wellbeing draws heavily on the PEAQ (Waterman, 1993), which has been shown to have good reliability and validity (Lee and Jeong, 2021; Park and Ahn, 2022; Su et al., 2020). The six items of the trait gratitude scale were adapted from the loss/adversity subscale in Adler and Fagley's (2005) Appreciation Scale, while the three items of the state gratitude scale were derived from Emmons and McCullough (2003). The five items measuring subjective wellbeing were drawn from Diener et al.'s (1985) Life Satisfaction Scale, and the six items used to measure psychological wellbeing were derived from the Psychological Wellbeing Scale developed by Ryff and Keyes (1995). The final section of the questionnaire collected respondents' basic information.

#### 5.3.2 Data collection and analysis

This study conducted an online pre-survey between April and May 2023 to assess the reliability and validity of the scales and ensure the scientific rigor of the formal survey. Employing snowball sampling, a total of 202 responses were collected. Invalid responses were excluded based on the following criteria: (1) abnormally short or long completion times; (2) straight-lining, patterned responses, or logical inconsistencies; and (3) failure to pass attention-check items. After data cleaning, 170 valid responses were retained for subsequent analysis.

Prior to conducting exploratory factor analysis (EFA), item analysis was first performed on the newly developed meaningful tourism experience scale. This procedure involved assessing the corrected item-total correlations (CITC) and evaluating the discriminative power of each item by comparing the top and bottom 27% of total scores using independent-samples *t*-tests. Two items with CITC values below the recommended threshold of 0.4 were removed (see Table 2). All remaining items demonstrated significant differences between the high and low scoring groups (*p* < 0.001). Furthermore, EFA of the refined scale indicated that all factor loadings and communalities exceeded 0.50, explaining 73.62% of the cumulative variance. Finally, EFA conducted on the entire questionnaire confirmed that all scales had KMO values exceeding 0.70, a significant Bartlett's test of sphericity, and Cronbach's alpha coefficients above 0.70, thereby demonstrating satisfactory overall reliability and validity.

**Table 2:** Results of item analysis and EFA for the Meaningful Tourism Experience Scale (*N* = 170).

**Measured item**	**CITC**	**Cronbach's alpha if item deleted**	**Dimension**	**Factor loading**	**% of variance**	**α**
P1: During this trip, I felt joyful.	0.611	0.926	Pleasure	0.900	17.695	0.901
P2: I felt delighted.	0.608	0.926		0.657		
P3: I felt relaxed.	0.522	0.928		0.874		
P4: I felt refreshed.	0.666	0.925		0.846		
G1: I felt my thoughts deepen.	0.530	0.927	Growth	0.542	12.873	0.790
G2: I felt a positive shift in my mindset.	0.596	0.926		0.724		
G3: I felt my skills improve.	0.723	0.924		0.570		
G4: I experienced growth and change.	0.572	0.927		0.773		
F1: I was able to do things I enjoy.	0.720	0.924	Freedom	0.602	15.555	0.905
F2: I felt liberated from the constraints of daily life.	0.678	0.925		0.855		
F3: I felt my true self being unleashed.	0.730	0.924		0.809		
F4: I felt truly free.	0.688	0.925		0.781		
F5: I was free to make my own choices. ^*^	0.380	0.931				
M1: I created some wonderful memories.	0.526	0.928	Creating memories	0.839	12.404	0.816
M2: I experienced some unforgettable moments.	0.649	0.926		0.703		
M3: I gathered some memorable travel experiences.	0.638	0.926		0.744		
M4: I was able to recall and feel that life was rich and colorful.	0.705	0.925		0.611		
M5: When I look back on this trip, I will feel that my time was well spent. ^*^	0.346	0.931				
E1: I contemplated the meaning of life.	0.485	0.928	Examining life	0.689	15.097	0.854
E2: I reflected on what truly matters in life.	0.550	0.927		0.775		
E3: I pondered how to achieve balance between life and work.	0.543	0.927		0.812		
E4: I considered how to lead a better, happier life.	0.630	0.926		0.839		

^*^Indicates items that were deleted.

To avoid common method bias, this study used both online and field research to conduct the formal survey. The online survey was primarily conducted through Wenjuanxing, a well-known data collection platform, while the field survey took place at the Wuhou Shrine, a popular tourist attraction in Chengdu, China. A total of 500 online and 350 on-site questionnaires were collected. Data cleaning followed the procedures established in the pilot study, with one additional criterion applied to the on-site data: questionnaires containing missing items were excluded. After data screening, 428 valid online and 313 valid on-site responses were included, yielding a final sample of 714 for the formal analysis. Table 3 presents the demographic profile of the respondents. SmartPLS 4 software was used to conduct confirmatory factor analysis (CFA) on the questionnaire scales and examine the relationships between the variables.

**Table 3:** Demographic profile of respondents.

**Group**	**Category**	**Frequency**	**Percentage**	**Group**	**Category**	**Frequency**	**Percentage**
Gender Education Age	Male	308	41.6	Marital status	Married	439	59.3
	Female	433	58.4		Unmarried	287	38.7
	High school or below	45	6.1		Others	15	2
	Diploma	104	14	Monthly income (CNY)	Below CNY 3,000	245	33.1
	Bachelor's degree	381	51.4		CNY 3,001–6,000	158	21.3
	Master's degree	182	24.6		CNY 6,001–10,000	170	23
	Doctoral degrees	29	3.9		CNY 10,000–20,000	124	16.7
	18–25	285	38.5		CNY 20,001 or above	44	5.9
	26–30	181	24.4	Occupation	Student	246	33.2
	31–40	176	23.8		Working staff	284	38.3
	41–60	81	10.9		Freelancer	75	10.1
	61 or older	18	2.4		Retiree	22	3
					Others	114	15.4

### 5.4 Results

#### 5.4.1 Confirmatory factor analysis of the meaningful tourism experience scale

A CFA was conducted on the 20-item meaningful tourism experience scale, with the results summarized in Table 4. The standardized factor loadings of all items on their respective predefined dimensions exceeded the recommended threshold of 0.70. The composite reliability (CR) values for each dimension ranged from 0.919 to 0.962, and all Cronbach's alpha coefficients exceeded the 0.70 threshold, indicating excellent internal consistency. Furthermore, the average variance extracted (AVE) for each dimension ranged from 0.74 to 0.86, all exceeding the 0.50 criterion, thereby supporting strong convergent validity.

**Table 4:** CFA results of meaningful tourism experiences.

**Dimension**	**Item**	**Loading**	**α**	**CR**	**AVE**	**MSV**	**ASV**
Examining life	E1	0.896	0.919	0.922	0.749	0.457	0.297
	E2	0.898					
	E3	0.804					
	E4	0.861					
Freedom	F1	0.817	0.917	0.927	0.756	0.475	0.396
	F2	0.872					
	F3	0.885					
	F4	0.901					
Growth	G1	0.843	0.915	0.919	0.740	0.457	0.378
	G2	0.880					
	G3	0.839					
	G4	0.878					
Pleasure	P1	0.930	0.950	0.962	0.863	0.536	0.339
	P2	0.952					
	P3	0.895					
	P4	0.939					
Creating memories	M1	0.927	0.951	0.956	0.846	0.536	0.417
	M2	0.923					
	M3	0.934					
	M4	0.895					

As shown in Table 4, both the average shared variance (ASV) and maximum shared variance (MSV) for each construct were lower than their respective AVE values, collectively demonstrating satisfactory discriminant validity across the five dimensions. The model fit indices for the five-factor model were χ^2^/*df* = 4.819, CFI = 0.957, TLI = 0.950, RMSEA = 0.072, and SRMR = 0.058. In summary, the five-factor, 20-item measurement model demonstrated a good fit to the data and exhibited robust reliability, convergent validity, and discriminant validity, supporting its use for subsequent hypothesis testing.

#### 5.4.2 Measurement model evaluation

Following validation of the five-factor structure, item parceling (Little et al., 2013) was employed to simplify the structural model. Specifically, items within each dimension were averaged to create five parcel scores, which served as reflective indicators of the higher-order construct of meaningful tourism experience in subsequent analyses. The results of the measurement model evaluation are presented in Table 5.

**Table 5:** Measurement model analysis results.

**Latent variables**	**Items**	**Loadings**	**α**	**CR**	**AVE**	**MSV**	**ASV**
Meaningful tourism experience	Examining life	0.720	0.849	0.855	0.624	0.578	0.486
	Freedom	0.820					
	Growth	0.782					
	Pleasure	0.772					
	Creating memories	0.851					
Eudaimonic tourism wellbeing	ETW1	0.955	0.898	0.899	0.907	0.578	0.397
	ETW2	0.950					
Hedonic tourism wellbeing	HTW1	0.917	0.765	0.780	0.809	0.549	0.361
	HTW2	0.881					
State gratitude	SG1	0.928	0.909	0.909	0.846	0.529	0.403
	SG2	0.929					
	SG3	0.902					
Trait gratitude	TG1	0.782	0.906	0.907	0.681	0.417	0.307
	TG2	0.813					
	TG3	0.825					
	TG4	0.848					
	TG5	0.838					
	TG6	0.842					
Subjective wellbeing	SWB1	0.830	0.886	0.887	0.686	0.514	0.414
	SWB2	0.843					
	SWB3	0.826					
	SWB4	0.815					
	SWB5	0.828					
Psychological wellbeing	PWB1	0.809	0.906	0.907	0.682	0.484	0.330
	PWB2	0.850					
	PWB3	0.882					
	PWB4	0.841					
	PWB5	0.839					
	PWB6	0.725					

All latent variables exhibited satisfactory reliability, with both Cronbach's alpha and CR values exceeding the 0.70 threshold. Convergent validity was established, as all indicator loadings were significant and exceeded 0.70, and the AVE for each construct surpassed the 0.50 criterion. Discriminant validity was also established, as shown in Table 5. In summary, the measurement model demonstrated satisfactory psychometric properties, providing a solid foundation for subsequent structural model analyses.

#### 5.4.3 Structural model evaluation

Results indicated that the variance inflation factor (VIF) values for all variables were below 5, suggesting that multicollinearity is not a concern in this study (Joseph et al., 2009). As Table 6 shows, the R^2^ values of all endogenous variables reached the medium-strength level, indicating that the model has good explanatory power. Furthermore, the Q^2^ values used to test the predictive relevance of the structural model were all greater than zero, demonstrating the model's strong predictive ability (Hair et al., 2011). In Table 6, the goodness-of-fit metric (GoF) is 0.646, which is greater than 0.36, indicating that the model is a good fit (Wetzels et al., 2009).

**Table 6:** Assessment of structural model.

**Endogenous variables**	**Communality**	***R* ^2^**	***Q* ^2^**	**GoF**
Eudaimonic tourism wellbeing	0.907	0.578	0.519	0.646
Hedonic tourism wellbeing	0.809	0.550	0.437	
State gratitude	0.846	0.591	0.496	
Psychological wellbeing	0.682	0.390	0.275	
Subjective wellbeing	0.686	0.543	0.367	

#### 5.4.4 Evaluation of direct and indirect effects

The hypothesized relationships were tested using a bootstrapping procedure with 5,000 resamples in SmartPLS 4. The results of the path analysis, including the direct, indirect, and total effects, are presented in Table 7. Overall, the model demonstrated strong empirical support. As shown in Table 7, all direct-effect hypotheses (H1a, H1b, H4, H5a, H5b, H6) were supported. Regarding the mediation hypotheses, five of the six proposed relationships were supported. Specifically, eudaimonic tourism wellbeing mediated the effects of meaningful tourism experience on both subjective and psychological wellbeing (H2a, H2b). Hedonic tourism wellbeing mediated the effect of meaningful tourism experience on subjective wellbeing (H3a). In addition, state gratitude mediated the relationships involving both forms of wellbeing (H7a, H7b). The only unsupported hypothesis was H3b, which posited that hedonic tourism wellbeing would mediate the relationship between meaningful tourism experience and psychological wellbeing (β = 0.063, *p* > 0.05).

**Table 7:** Results of hypothesis tests.

**Hypothesis**	**Direct paths**	**Standard coefficient**	***T*-value**	***P*-value**	**Result**
H1a	MTE → ETW	0.760	34.637	0.000	Support
H1b	MTE → HTW	0.741	32.164	0.000	Support
H4	MTE → SG	0.531	13.321	0.000	Support
H5a	TG → SWB	0.135	3.157	0.002	Support
H5b	TG → PWB	0.159	3.479	0.001	Support
H6	TG → SG	0.317	7.421	0.000	Support
**Indirect paths**					
H2a	MTE → ETW → SWB	0.213	6.063	0.000	Support
H2b	MTE → ETW → PWB	0.238	5.964	0.000	Support
H3a	MTE → HTW → SWB	0.156	4.377	0.000	Support
H3b	MTE → HTW → PWB	0.063	1.794	0.073	Reject
H7a	MTE → SG → SWB	0.134	5.155	0.000	Support
H7b	MTE → SG → PWB	0.111	3.906	0.000	Support
**Total effects**					
	MTE → SWB	0.503	13.859	0.000	
	MTE → PWB	0.413	10.711	0.000	
	TG → SWB	0.215	5.182	0.000	
	TG → PWB	0.225	5.090	0.000	

MTE, meaningful tourism experience; ETW, eudaimonic tourism wellbeing; HTW, hedonic tourism wellbeing; SG, state gratitude; TG, trait gratitude; SWB, subjective wellbeing; PWB, psychological wellbeing.

## 6 Conclusions and implications

This study sought to elucidate the profound impact of traumatic events on tourists' psychology and tourism experiences and explore how posttraumatic meaningful tourism experiences can help individuals seek—and enrich their conception of—meaning in life, promote transformation, and ultimately enhance their wellbeing. To this end, this study conducted two sub-studies, the combined results of which can be summarized as follows. First, reflective thematic analysis clarified the trauma and transformation experienced by tourists following the COVID-19 pandemic. Specifically, the experience of uncertainty seriously threatened travelers' needs for safety, social connection, and life coherence. It disrupted their sense of control and predictability, triggering feelings of anxiety, worry, and fear. Pandemic fatigue caused by persistent and cumulative stress exacerbated these negative emotions, resulting in physical fatigue and emotional exhaustion. In this respect, the trauma awakened an understanding of the dual nonbeing consciousness of body and mind, causing tourists to experience existential anxiety about death, loneliness, and meaninglessness.

In terms of transformation, the findings of this study showed that struggling with traumatic events can increase individuals' gratitude for the positive aspects of life and help reshape their perceptions of difficulties and suffering. Contemplation on significance and heightened self-awareness can assist tourists in attaining clarity regarding the essence of life and its purpose. This process offers a chance to explore and reconstruct the significance of life. Therefore, as predicted by Miao et al. (2021), in the post-COVID-19 era, people are likely to view travel as a means of pursuing meaning and alleviating their sense of existential crisis. In addition, it was found that the pandemic trauma prompted tourists to look more closely at the importance of tourism activities and realize their contribution to wellbeing.

Second, employing a mixed research approach, this study identified five dimensions of posttraumatic meaningful tourism experiences—namely, pleasure, freedom, growth, creating memories, and examining life—and developed a 20-item scale to measure meaningful tourism experiences. As demonstrated in prior research, pleasure constitutes an important source of life meaning (King and Hicks, 2021). Pleasurable tourism experiences substantively support and enhance life meaning by fostering positive emotional states. Experiences of freedom allow tourists to break away from everyday constraints, satisfy their need for autonomy, and create opportunities to connect with their authentic selves. The emergence of this dimension is closely associated with crisis-related experiences, which distinguishes it from the findings of previous meaningful tourism research (Chirakranont and Sakdiyakorn, 2022). In this context, growth reflects how adversity and learning within tourism experiences facilitate personal transformation and development, thereby serving as a crucial foundation for enriching life meaning. Through creating memories, tourism facilitates the appreciation of life's coherence, purpose, and significance. This shared or cherished memory-making process directly enhances individuals' sense of meaning in life. Finally, examining life represents a highly cognitive, reflective process through which tourists can deconstruct and reconstruct their understanding of life, enabling profound meaning reconstruction and transformation. This aligns with the view of reflection as a core mechanism for fostering meaningful experiences (Bastiaansen and Duerden, 2025). This study represents the first empirical validation of the dimensional structure of meaningful tourism experiences within a post-crisis context.

Third, this study demonstrated that meaningful tourism experiences can influence overall wellbeing by affecting tourism wellbeing and state gratitude. These findings support the bottom-up theory, which suggests that positive or enjoyable moments contribute to wellbeing (Diener and Ryan, 2009; Sirgy, 2019). However, regarding psychological wellbeing, the mediating effect of hedonic tourism wellbeing was not significant in this study. This result highlights the asymmetry in the impact of tourism wellbeing on overall wellbeing. The finding aligns with research showing that both eudaimonic and hedonic tourism wellbeing contribute to subjective wellbeing (Park and Ahn, 2022), whereas improvements in psychological wellbeing are more closely associated with activities involving personal meaning and self-growth (Su et al., 2020). In addition, it was found that meaningful tourism experiences can elicit gratitude in tourists, which further augments overall wellbeing. Thus, by integrating gratitude into the tourism wellbeing model, this study advances existing theory by empirically confirming the interconnected roles of meaningful experiences, gratitude, and wellbeing, as established in prior psychological research (Emmons and McCullough, 2003; Walker et al., 2016).

Finally, the results indicated that gratitude is an important characteristic influencing tourists' overall wellbeing. Tourists with high trait gratitude typically exhibit higher levels of subjective and psychological wellbeing. The empirical evidence in this study corroborates previous findings regarding the positive role of trait gratitude in psychological wellbeing (Măirean et al., 2019). In addition to direct effects, trait gratitude can influence state gratitude during tourism experiences, thereby enhancing wellbeing. Notably, although gratitude is widely recognized in psychology as a key trait that promotes wellbeing (Watkins et al., 2003; Wood et al., 2009), research within tourism studies has been limited in examining the relationship between trait gratitude and wellbeing (Filep et al., 2017). By incorporating trait gratitude, this study enriches the conclusions of bottom-up theory while expanding the current literature on tourists' gratitude.

### 6.1 Theoretical implications

This study contributes to the existing literature in the following ways. First, this study examined the effects of cumulative traumatic events on tourists' psychology and travel experiences, thereby enriching the research on the indirect and long-term effects of major traumatic events. Significantly, this study embedded the COVID-19 pandemic into the broader human experience, providing a better understanding and prediction of the psychology and behavior of tourists following other traumatic events.

Second, the study identified the dimensions of meaningful tourism experiences after trauma and developed a measurement scale. In doing so, this study contributes toward expanding theoretical frameworks for examining tourism experiences and lays the foundation for future research and evaluations regarding meaningful tourism experiences. While some scholars have suggested that travel can facilitate experiences of life meaning, there is relatively little research on meaningful tourism experiences as a pathway to post-trauma transformation. Furthermore, there is a lack of consensus on the elements involved in awakening meaning within tourism experiences. Therefore, research on meaningful tourism experiences with transformative potential following trauma is crucial to address this gap in the literature.

Third, this study's findings regarding the mediating role of tourism wellbeing and its asymmetric impact on overall wellbeing further clarify how posttraumatic tourism experiences can influence individuals' overall wellbeing. Accordingly, this study provides valuable insights for current research on tourism experiences and tourism-related subjective and psychological wellbeing in the context of adversity and trauma.

Finally, this study confirmed the impact of traumatic events on tourists' gratitude and adopted a top-down theoretical perspective to investigate the influence of positive personality traits on wellbeing. In addition to expanding the body of research on tourist gratitude after trauma, this study deepens the application of top-down theories of wellbeing in the tourism field. Scholars have tended to examine the impact of tourism experience on wellbeing from a bottom-up theoretical perspective, neglecting the spontaneous impacts of tourists' stable personality traits. This study confirmed that meaningful tourism experiences influence overall wellbeing through state gratitude, providing scholars with a new perspective to explore the relationship between experience and wellbeing, namely, through the lens of tourists' gratitude.

### 6.2 Practical implications

First, transformative experiences are increasingly becoming the core of the experience economy and are favored by consumers. The findings of this study provide practical guidelines for destinations and tourism companies to consciously design meaningful tourism experiences to support tourists' transformation. Destination managers can systematically develop tourism products based on the five dimensions identified in this study. For instance, they may design programs that promote personal growth, such as handicraft workshops or ecological conservation initiatives; create meditation spaces and tranquil walking trails that encourage visitors to reflect on life; and incorporate elements of surprise or serendipity to enhance the memorability of tourists' experiences. In terms of marketing, emphasizing the meaningful attributes and transformative potential of tourism experiences, as well as articulating their desired transformative outcomes, can help attract potential tourists.

Second, this study's findings suggest that meaningful tourism experiences can enhance tourist wellbeing by promoting state gratitude. Therefore, tourism practitioners and managers must guide tourists to engage in gratitude practices during tourism activities. Examples include offering “gratitude journals” in accommodation settings to encourage reflective writing; training tour guides to facilitate shared appreciation of natural or cultural encounters; and designing volunteer tourism components that emphasize reciprocal gratitude between visitors and host communities. Third, this study found that eudaimonic tourism wellbeing contributes to psychological and subjective wellbeing, while hedonic tourism wellbeing only enhances subjective wellbeing. Policymakers should prioritize investments and promotional efforts in tourism forms that facilitate self-discovery, continuous learning, and the pursuit of personal meaning. Establishing certification schemes or funding programs for community-based cultural and educational ecotourism could help channel market demand toward experiences that deliver deeper and more holistic wellbeing benefits.

Finally, this study revealed that trait gratitude may change after trauma and validated its positive effect on wellbeing, offering new insights for improving post-trauma psychological assistance. Social psychological service departments can guide affected tourists to view their trauma from a dialectical perspective, helping them reevaluate their traumatic experiences and rebuild core beliefs. By encouraging individuals to confront adversity positively and cultivate a grateful personality, this approach can help them move toward improved wellbeing and a flourishing life.

### 6.3 Limitations and paths for future research

This study acknowledges several limitations. First, the dimensions and specific connotations of meaningful tourism experiences may vary across cultures. In the future, researchers should study and compare the differences and connections between the connotations and dimensions of meaningful tourism experiences in different cultures, especially with regard to their unique characteristics. Second, although this study endeavored to combine bottom-up and top-down theories, the personality trait variables only considered the role of gratitude. As such, further exploration of the effects of other tourist personality traits, such as extraversion, agreeableness, and optimism, on wellbeing is warranted. Finally, another area that deserves further exploration is how personality traits conducive to a good life can be fostered through tourism.

## Data Availability

The raw data supporting the conclusions of this article will be made available by the authors, without undue reservation.

## References

[B1] Adler M. G. Fagley N. S. (2005). Appreciation: individual differences in finding value and meaning as a unique predictor of subjective well-being. J. Personal. 73, 79–114. doi: 10.1111/j.1467-6494.2004.00305.x 15660674

[B2] Bastiaansen M. Duerden M. D. (2025). Conceptualizing Meaningful Experiences. J. Hosp. Tour. Res. 49, 877–890. doi: 10.1177/10963480241308344

[B3] Bhalla R. Chowdhary N. Ranjan A. (2021). Spiritual tourism for psychotherapeutic healing post COVID-19. J. Travel Tour. Market. 38, 769–781. doi: 10.1080/10548408.2021.1930630

[B4] Bosangit C. Hibbert S. McCabe S. (2015). “If I was going to die I should at least be having fun”: Travel blogs, meaning and tourist experience. Ann. Tour. Res. 55, 1–14. doi: 10.1016/j.annals.2015.08.001

[B5] Braun V. Clarke V. (2006). Using thematic analysis in psychology. Qual. Res. Psychol. 3, 77–101. doi: 10.1191/1478088706qp063oa

[B6] Braun V. Clarke V. (2019). Reflecting on reflexive thematic analysis. Qual. Res. Sport Exer. Health 11, 589–597. doi: 10.1080/2159676X.2019.1628806

[B7] Braun V. Clarke V. (2021). To saturate or not to saturate? Questioning data saturation as a useful concept for thematic analysis and sample-size rationales. Qual. Res. Sport Exerc. Health 13, 201–216. doi: 10.1080/2159676X.2019.1704846

[B8] Brown L. (2013). Tourism: a catalyst for existential authenticity. Ann. Tour. Res. 40, 176–190. doi: 10.1016/j.annals.2012.08.004

[B9] Buckley R. Westaway D. (2020). Mental health rescue effects of women's outdoor tourism: a role in COVID-19 recovery. Ann. Tour. Res. 85:103041. doi: 10.1016/j.annals.2020.103041 33100433 PMC7575266

[B10] Câmara E. Pocinho M. Agapito D. Jesus S. N. D. (2023). Meaningful experiences in tourism: a systematic review of psychological constructs. Eur. J. Tour. Res. 34:3403. doi: 10.54055/ejtr.v34i.2964

[B11] Cheng L. Liu L. (2022). Exploring posttraumatic growth after the COVID-19 pandemic. Tour. Manag. 90:104474. doi: 10.1016/j.tourman.2021.104474 34924667 PMC8664663

[B12] Chirakranont R. Sakdiyakorn M. (2022). Conceptualizing meaningful tourism experiences: case study of a small craft beer brewery in Thailand. J. Dest. Market. Manag. 23:100691. doi: 10.1016/j.jdmm.2022.100691

[B13] Churchill G. A. (1979). A paradigm for developing better measures of marketing constructs. J. Market. Res. 16, 64–73. doi: 10.1177/002224377901600110

[B14] Creswell J. W. Clark V. L. P. (2018). Designing and conducting mixed methods research (third edition). Washington DC: Sage Publications.

[B15] Decrop A. (1999). Triangulation in qualitative tourism research. Tour. Manag. 20, 157–161. doi: 10.1016/S0261-5177(98)00102-2

[B16] DeVellis R. F. (2017). Scale Development: Theory and Applications. Washington DC: Sage.

[B17] Diener E. Emmons R. A. Larsen R. J. Griffin S. (1985). The satisfaction with life scale. J. Person. Assess. 49, 71–75. doi: 10.1207/s15327752jpa4901_13 16367493

[B18] Diener E. Oishi S. Lucas R. E. (2003). Personality, culture, and subjective well-being: emotional and cognitive evaluations of life. Ann. Rev. Psychol. 54, 403–425. doi: 10.1146/annurev.psych.54.101601.145056 12172000

[B19] Diener E. Ryan K. (2009). Subjective well-being: a general overview. South Afr. J. Psychol. 39, 391–406. doi: 10.1177/008124630903900402

[B20] Diner E. (1984). Subjective well-being. Psychol. Bull. 95, 542–575. doi: 10.1037/0033-2909.95.3.542 6399758

[B21] Emmons R. A. McCullough M. E. (2003). Counting blessings versus burdens: an experimental investigation of gratitude and subjective well-being in daily life. J. Personal. Social Psychol. 84, 377–389. doi: 10.1037/0022-3514.84.2.377 12585811

[B22] Filep S. Macnaughton J. Glover T. (2017). Tourism and gratitude: valuing acts of kindness. Ann. Tour. Res. 66, 26–36. doi: 10.1016/j.annals.2017.05.015

[B23] Filep S. Moyle B. D. Skavronskaya L. (2022). Tourist wellbeing: re-thinking hedonic and eudaimonic dimensions. J. Hosp. Tour. Res. 1467013183. doi: 10.1177/10963480221087964

[B24] Frankl V. E. (1992). Man's search for meaning: an introduction to logotherapy. 4th Edn. New York: Simon and Schuster.

[B25] George L. S. Park C. L. (2016). Meaning in life as comprehension, purpose, and mattering: toward integration and new research questions. Rev. Gen. Psychol. 20, 205–220. doi: 10.1037/gpr0000077

[B26] Grimwood B. S. R. Lopez K. J. Leighton J. Stevens Z. (2023). Practices of gratitude and outdoor leisure. Leisure Sci. 47, 1–24. doi: 10.1080/01490400.2023.2206394

[B27] Hair J. F. Ringle C. M. Sarstedt M. (2011). PLS-SEM: indeed a silver bullet. J. Market. Theory Pract. 19, 139–152. doi: 10.2753/MTP1069-6679190202

[B28] Huang X. Wang P. Wu L. (2023). Well-being through transformation: an integrative framework of transformative tourism experiences and hedonic versus eudaimonic well-being. J. Travel Res. 63, 974–994. doi: 10.1177/00472875231171670

[B29] Huta V. Waterman A. S. (2014). Eudaimonia and its distinction from hedonia: developing a classification and terminology for understanding conceptual and operational definitions. J. Happ. Stud. 15, 1425–1456. doi: 10.1007/s10902-013-9485-0

[B30] Iwasaki Y. (2008). Pathways to meaning-making through leisure-like pursuits in global contexts. J. Leisure Res. 40, 231–249. doi: 10.1080/00222216.2008.11950139

[B31] Jain S. Banerjee R. Sharma R. W. (2023). Meaning-oriented consumption: a systematic review and research agenda. Int. J. Cons. Stud. 47, 2305–2334. doi: 10.1111/ijcs.12927

[B32] Janoff-Bulman R. (1992). Shattered assumptions. Maxwell Macmillan Canada, Inc.

[B33] Joseph F. Hair J. William C. Black Barry J. Babin Rolph E. Anderson. (2009). Multivariate Data Analysis (7th Edn.). Pearson New International Edition.

[B34] Kay Smith M. Diekmann A. (2017). Tourism and wellbeing. Ann. Tour. Res. 66, 1–13. doi: 10.1016/j.annals.2017.05.006

[B35] Kim S. Lee J. S. (2013). Is satisfaction enough to ensure reciprocity with upscale restaurants? The role of gratitude relative to satisfaction. Int. J. Hosp. Manage. 33, 118–128. doi: 10.1016/j.ijhm.2012.06.009

[B36] King L. A. Hicks J. A. (2021). The science of meaning in life. Ann. Rev. Psychol. 72, 561–584. doi: 10.1146/annurev-psych-072420-122921 32898466

[B37] Kirillova K. Lehto X. (2015). An existential conceptualization of the vacation cycle. Ann. Tour. Res. 55, 110–123. doi: 10.1016/j.annals.2015.09.003

[B38] Kirillova K. Lehto X. Cai L. (2017a). Tourism and existential transformation: an empirical investigation. J. Travel Res. 56, 638–650. doi: 10.1177/0047287516650277

[B39] Kirillova K. Lehto X. Cai L. (2017b). Existential authenticity and anxiety as outcomes: the tourist in the experience economy. Int. J. Tour. Res. 19, 13–26. doi: 10.1002/jtr.2080

[B40] Lee W. Jeong C. (2021). Distinctive roles of tourist eudaimonic and hedonic experiences on satisfaction and place attachment: combined use of SEM and necessary condition analysis. J. Hosp. Tour. Manage. 47, 58–71. doi: 10.1016/j.jhtm.2021.02.012

[B41] Little T. D. Rhemtulla M. Gibson K. Schoemann A. M. (2013). Why the items versus parcels controversy needn't be one. Psychol. Met. 18, 285–300. doi: 10.1037/a0033266 23834418 PMC3909043

[B42] Liu L. Cheng L. Qu X. (2023). From existential anxiety to posttraumatic growth: The stranded traveler during the pandemic outbreak. Ann. Tour. Res. 99:103548. doi: 10.1016/j.annals.2023.103548 36936515 PMC10000268

[B43] Măirean C. Turliuc M. N. Arghire D. (2019). The relationship between trait gratitude and psychological wellbeing in university students: the mediating role of affective state and the moderating role of state gratitude. J. Happines. Stud. 20, 1359–1377. doi: 10.1007/s10902-018-9998-7

[B44] Malterud K. Siersma V. D. Guassora A. D. (2016). Sample size in qualitative interview studies: guided by information power. Qual. Health Res. 26, 1753–1760. doi: 10.1177/1049732315617444 26613970

[B45] Martela F. Steger M. F. (2016). The three meanings of meaning in life: Distinguishing coherence, purpose, and significance. J. Posit. Psychol. 11, 531–545. doi: 10.1080/17439760.2015.1137623

[B46] Miao L. Im J. Fu X. Kim H. Zhang Y. E. (2021). Proximal and distal post-COVID travel behavior. Ann. Tour. Res. 88:103159. doi: 10.1016/j.annals.2021.103159

[B47] Milazzo L. Soulard J. (2024). Bridging disciplinary perspectives on transformation: epistemologically evaluating liminality and transformative learning. Ann. Tour. Res. 104:103710. doi: 10.1016/j.annals.2023.103710

[B48] Neuhofer B. (2024). Transformative experiences: a conceptual analysis of the integration process. Serv. Industr. J. 44, 522–537. doi: 10.1080/02642069.2024.2342295

[B49] Park S. Ahn D. (2022). Seeking pleasure or meaning? the different impacts of hedonic and eudaimonic tourism happiness on tourists' life satisfaction. Int. J. Environ. Res. Public Health 19:1162. doi: 10.3390/ijerph19031162 35162186 PMC8834700

[B50] Peng J. Yang X. Fu S. Huan T. T. C. (2023). Exploring the influence of tourists' happiness on revisit intention in the context of Traditional Chinese Medicine cultural tourism. Tour. Manag. 94:104647. doi: 10.1016/j.tourman.2022.104647

[B51] Pung J. M. Gnoth J. Del Chiappa G. (2020). Tourist transformation: Towards a conceptual model. Ann. Tour. Res. 81:102885. doi: 10.1016/j.annals.2020.102885

[B52] Ryan R. M. Deci E. L. (2001). On happiness and human potentials: a review of research on hedonic and eudaimonic well-being. Ann. Rev.Psychol. 52, 141–166. doi: 10.1146/annurev.psych.52.1.141 11148302

[B53] Ryff C. (1989). Happiness is everything, or is it - explorations on the meaning of psychological well-being. J. Pers. Soc. Psychol. 57, 1069–1081. doi: 10.1037/0022-3514.57.6.1069

[B54] Ryff C. D. Keyes C. (1995). The structure of psychological well-being revisited. J. Pers. Soc. Psychol. 69, 719–727. doi: 10.1037/0022-3514.69.4.719 7473027

[B55] Sengupta S. (2022). Travel after tragedy: A phenomenological study on what it takes for women to travel solo after tragedy. J. Leisure Res. 53, 92–111. doi: 10.1080/00222216.2021.1899777

[B56] Sheldon P. J. (2020). Designing tourism experiences for inner transformation. Ann. Tour. Res. 83:102935. doi: 10.1016/j.annals.2020.102935

[B57] Sirgy J. M. (2019). Promoting quality-of-life and well-being research in hospitality and tourism. J. Travel Tour. Market. 36, 1–13. doi: 10.1080/10548408.2018.1526757

[B58] Steger M. F. Oishi S. Kesebir S. (2011). Is a life without meaning satisfying? The moderating role of the search for meaning in satisfaction with life judgments. J. Posit. Psychol. 6, 173–180. doi: 10.1080/17439760.2011.569171

[B59] Su L. Tang B. Nawijn J. (2020). Eudaimonic and hedonic well-being pattern changes: Intensity and activity. Ann. Tour. Res. 84:103008. doi: 10.1016/j.annals.2020.103008

[B60] Su L. Tang B. Nawijn J. (2021). How tourism activity shapes travel experience sharing: Tourist well-being and social context. Ann. Tour. Res. 91:103316. doi: 10.1016/j.annals.2021.103316

[B61] Tedeschi R. G. Calhoun L. G. (2004). Posttraumatic growth: conceptual foundations and empirical evidence. Psychol. Inq. 15, 1–18. doi: 10.1207/s15327965pli1501_01

[B62] Tedeschi R. G. Shakespeare-Finch J. Taku K. Calhoun L. G. (2018). Posttraumatic growth: theory, research, and applications (1st Edn.). New York: Routledge.

[B63] Tu H. Ma J. (2021). Does positive contact between residents and tourists stimulate tourists' environmentally responsible behavior? The role of gratitude and boundary conditions. J. Travel Res. 6882407. doi: 10.1177/00472875211048938

[B64] Vada S. Prentice C. Scott N. Hsiao A. (2020). Positive psychology and tourist well-being: A systematic literature review. Tour. Manag. Perspect. 33:100631. doi: 10.1016/j.tmp.2019.100631

[B65] Walker J. Kumar A. Gilovich T. (2016). Cultivating gratitude and giving through experiential consumption. Emotion 16, 1126–1136. doi: 10.1037/emo0000242 27797561

[B66] Waterman A. S. (1993). Two conceptions of happiness: contrasts of personal expressiveness (eudaimonia) and hedonic enjoyment. J. Pers. Soc. Psychol. doi: 10.1037/0022-3514.64.4.678

[B67] Watkins P. C. Woodward K. Stone T. Kolts R. L. (2003). Gratitude and happiness: development of a measure of gratitude, and relationships with subjective well-being. Soc. Behav. Pers. An Int. J. 31, 431–451. doi: 10.2224/sbp.2003.31.5.431

[B68] Wattanacharoensil W. Pattaratanakun A. Taecharungroj V. Solnet D. (2024). Toward an improved, holistic understanding of ‘meaningful destination'. Tour. Recreat. Res. 1–17. doi: 10.1080/02508281.2024.2357001

[B69] Westoby R. Clissold R. McNamara K. E. (2022). Hopeful tourism to grapple and engage with emotions in the Anthropocene. Geograph. Res. 60, 651–656. doi: 10.1111/1745-5871.12554

[B70] Wetzels M. Odekerken-Schröder G. van Oppen C. (2009). Using PLS path modeling for assessing hierarchical construct models: guidelines and empirical illustration. Mis. Quart. 33, 177–195. doi: 10.2307/20650284

[B71] Wong I. A. Lin Z. C. Yang F. X. Kou I. E. (2023). From shattered assumptions to fantasy realization: the role of meaning search in pleasure travel during the COVID era. Curr. Issues Tour. 1–19. doi: 10.1080/13683500.2023.2220954

[B72] Wong P. T. P. (2020). Made for resilience and happiness: effective coping with covid-19 according to Viktor E. Frankl and Paul T. P. Wong. Binghamton, NY: INPM Press.

[B73] Wood A. M. Joseph S. Maltby J. (2009). Gratitude predicts psychological well-being above the Big Five facets. Personal. Individ. Differ. 46, 443–447. doi: 10.1016/j.paid.2008.11.012

[B74] Wood A. M. Maltby J. Stewart N. Linley P. A. Joseph S. (2008). A social-cognitive model of trait and state levels of gratitude. Emotion 8, 281–290. doi: 10.1037/1528-3542.8.2.281 18410201

[B75] Yalom I. D. (1980). Existential psychotherapy. New York: Basic Books.

[B76] Zhang X. Cheng L. Ma G. (2024). Eliciting eudaimonic well-being in the tourism experiential space: Evidence from online reviews. Tour. Manag. 105:104955. doi: 10.1016/j.tourman.2024.104955

[B77] Zhuo S. Xu Y. Jiang T. (2024). What enables the effects of transformative tourism experiences to persist? Insights from identity development theory. Curr. Issues Tour. 28, 1–17. doi: 10.1080/13683500.2024.2374383

