# Supplementary material for: Meaningful tourism experiences and the cultivation of wellbeing effects: transformative practice of posttraumatic travel
Source: Front Psychol. 2026 Jan 30;17:1714606. doi: 10.3389/fpsyg.2026.1714606 (PMC12900676; doi:10.3389/fpsyg.2026.1714606)
Supplement: Supplementary file 1 [file Table_1.docx]

**Appendix A.**

Interview Outline

(Note: Interviewers are free to reorder questions or ask follow-up questions to explore certain topics in depth based on respondents’ answers.)

**Basic Information about Tourists**:

Name, age, education level, occupation, city of residence, etc.

**Impact of the Pandemic on Tourists’ Work, Lifestyle, and Travel as Well as the Transformations It Caused.**

- Do you currently worry about the possibility of another wave of the pandemic?
- Have you or anyone close to you been infected with COVID-19? Could you describe the situation?
- How has the repeated and prolonged pandemic over the past three years affected your life and work?
- What was the most significant impact the pandemic had on you?
- Did you travel during the pandemic? If so, could you describe your experience at that time?
- Compared to before the pandemic, do you think there have been any psychological changes in yourself now? Are there any positive changes?

**Tourism Choices and Experiences After the End of the Pandemic**

- How many trips have you taken since the pandemic ended? Where did you go?
- What kinds of activities did you experience during your travels?
- How did you feel during this trip compared to the trips you took before and during the pandemic (if any)?
- Were you satisfied with this trip? Why or why not?
- How did you feel after the trip?

**Tourists’ Interpretations of Meaningful Tourism Experiences That Contributed to Their Transformation**

- How do you understand the meaning in life?
- Is traveling important to you? Is it meaningful?
- What kind of tourism experiences do you find meaningful?
- What elements do you think a meaningful tourism experience should include? Could you provide specific examples?
- Do travel experiences that enrich the meaning in life bring you a sense of well-being? Why?

**Appendix B.**

**Table**. Themes and their connotations

| Themes | Sub-themes | Connotation of themes |
| --- | --- | --- |
| Uncertainty Experience | Uncertainty of the pandemic’s progression | Uncertainty experience reflected the various uncertainties tourists experienced in terms of potential losses and threats—including physical harm, mental health issues, wasted time, and financial crises—as well as the efforts made to cope with these uncertainties. |
|  | Uncertainty in life |  |
|  | Coping strategies to enhance a sense of control |  |
| Pandemic Fatigue | Information fatigue | Pandemic fatigue encompasses a range of negative physical, psychological, and behavioral responses triggered by prolonged exposure to the risks associated with the pandemic, the stress induced by containment measures, and the inherent conflict between the ongoing, unpredictable nature of the pandemic and the pervasive desire for its resolution. |
|  | Mental fatigue |  |
|  | Physical fatigue |  |
| Existential Anxiety | Death anxiety | Traumatic experiences during the COVID-19 pandemic—such as the threat of death, constant vigilance, and the loss of daily routines—exposed people to existential dilemmas, leading to existential anxiety related to death, loneliness, and meaninglessness among tourists. |
|  | Loneliness anxiety |  |
|  | Meaninglessness anxiety |  |
| Meaning-seeking | Shattered assumptions | Meaning-seeking refers to the desire to re-establish or deepen one’s perception and understanding of the meaning, value, and purpose of life, shaped by the psychological and existential triggers associated with the traumatic impact of the COVID-19 pandemic. |
|  | Reassessing the meaning in life |  |
|  | Increased self-awareness |  |
| Gratitude | Perceived social support | Gratitude reflects the recognition that, despite suffering and hardship during the COVID-19 trauma, travelers, through a deeper understanding of life’s meaning and value and through profound reflection and experience, come to cherish and appreciate what they have, as well as the beauty and preciousness of life. Enhanced gratitude can be observed in three levels and two types (state and trait): emotional responses elicited by perceiving support, help, and sacrifices from others (state gratitude); immediate emotional reactions of gratitude prompted by appreciation of aspects of life that are worthy of attention and care (state gratitude); and a broader life orientation trait, reflecting an appreciation and gratitude toward life, the world, and others (trait gratitude). |
|  | Appreciation of daily life |  |
|  | Tendency to cherish and be grateful |  |
|  | Prosocial behavior |  |
| Tourism well-being | Tourism memories | Tourism well-being is the sense of well-being experienced by travelers participating in tourism activities. The theme reflects the impact of factors such as travel restrictions during the pandemic and is further reinforced by travel memories, imperfect travel experiences during the COVID-19 pandemic, and perceptions of the importance of tourism. |
|  | Travel experiences during the pandemic |  |
|  | Tourism importance |  |
| Meaningful tourism experiences | Pleasure | Meaningful tourism experience refers to tourism experiences that offer tourists a sense of meaning in life—that is, experiences that inspire tourists to understand and appreciate the meaning of life and to cultivate their awareness of the purpose, mission, and significance of their own lives. |
|  | Freedom |  |
|  | Growth |  |
|  | Creating memories |  |
|  | Examining Life |  |
